# LAPAROSCOPIC APPENDICECTOMY: RISK FACTORS FOR CONVERSION TO LAPAROTOMY

**DOI:** 10.1590/0102-672020230019e1737

**Published:** 2023-06-02

**Authors:** Mouna Cherif, Meryam Mesbahi, Haithem Zaafouri, Helmi Zebda, Nizar Khedhiri, Dhafer Hadded, Anis Ben-Maamer

**Affiliations:** 1 Habib Thameur Hospital, Visceral Surgery – Tunis, Tunisia.

**Keywords:** Appendectomy, Laparoscopy, Conversion to open surgery, Laparotomy, Apendicectomia, Laparoscopia, Conversão para cirurgia aberta, Laparotomia

## Abstract

**BACKGROUND:**

Laparoscopic appendectomy is the gold standard surgical procedure currently performed for acute appendicitis. The conversion rate is one of the main factors used to measure laparoscopic competence, being important to avoid wasting time in a laparoscopic procedure and proceed directly to open surgery.

**AIMS:**

To identify the main preoperative parameters associated with a higher risk of conversion in order to determine the surgical method indicated for each patient.

**METHODS:**

Retrospective study of patients admitted with acute appendicitis who underwent laparoscopic appendectomy. A total of 725 patients were included, of which 121 (16.7%) were converted to laparotomy.

**RESULTS:**

The signiﬁcant factors that predicted conversion, identified by univariate and multivariate analysis, were: the presence of comorbidities (OR 3.1; 95%CI; p<0.029), appendicular perforation (OR 5.1; 95%CI; p<0.003), retrocecal appendix (OR 5.0; 95%CI; p<0.004), gangrenous appendix, presence of appendicular abscess (OR 3.6; 95%CI; p<0.023) and the presence of difficult dissection (OR 9.2; 95%CI; p<0.008).

**CONCLUSIONS:**

Laparoscopic appendectomy is a safe procedure to treat acute appendicitis. It is a minimally invasive surgery and has many advantages. Preoperatively, it is possible to identify predictive factors for conversion to laparotomy, and the ability to identify these reasons can aid surgeons in selecting patients who would beneﬁt from a primary open appendectomy.

## RESUMO

**RACIONAL:**

A apendicectomia laparoscópica é o procedimento cirúrgico padrão-ouro realizado atualmente para apendicite aguda. A taxa de conversão é um dos principais fatores utilizados para medir a competência laparoscópica, e importante para evitar perda de tempo em um procedimento laparoscópico e proceder diretamente à cirurgia aberta.

**OBJETIVO:**

Identiﬁcar os principais parâmetros pré-operatórios associados ao maior risco de conversão para determinar o método cirúrgico indicado para cada paciente.

**MÉTODOS:**

Estudo retrospectivo de pacientes admitidos com apendicite aguda, submetidos a apendicectomia laparoscópica. Foram incluídos 725 pacientes, sendo que destes, 121 (16,7%) foram convertidos para laparotomia.

**RESULTADOS:**

Os fatores signiﬁcativos que predizem a conversão, identiﬁcados por análise univariada e multivariada, foram: presença de comorbidades (OR 3,1; IC95%; p<0,029), perfuração apendicular (OR 5,1; IC95%; p<0,003), apêndice retrocecal (OR 5,0; IC95%; p<0,004), apêndice gangrenoso, presença de abscesso apendicular (OR 3,6; IC95%; p<0,023) e a presença de dissecção difícil (OR 9,2; IC95%; p<0,008).

**CONCLUSÕES:**

A apendicectomia laparoscópica é um procedimento seguro para tratar apendicite aguda. É uma cirurgia minimamente invasiva e tem muitas vantagens. No pré-operatório, é possível identificar os fatores preditores de conversão para laparotomia, e a capacidade de identificar essas razões pode ajudar os cirurgiões na seleção de pacientes que se beneficiariam de uma apendicectomia aberta primária.

## INTRODUCTION

Open appendectomy (OA) was considered the standard approach for acute appendicitis for a long time^7,10^. Laparoscopic appendectomy (LA) was reported for the ﬁrst time in 1983, and since then it has been adopted as the gold standard for the treatment of acute appendicitis^4,9,15^. For quite some time, its advantages have been evaluated by some authors in systematic reviews or meta-analysis^15,16^. Many studies conﬁrmed that the laparoscopic approach is a mainstream technique in acute appendicitis^3,7^. However, complicated appendicitis is considered a dilemma in their management regarding the greater demand for a high-quality experience and the fear of postoperative septic complications^15,16^. But the LA approach is moreover used in complicated patterns and rates of conversion to OA varies from 1 to 10%^6^. Therefore, conversion to OA may be the best solution in these cases^15^. A decision on which approach should be performed must be subject to preoperative criteria^1,10^.

We aimed to evaluate the predictors of conversion from LA to OA through a retrospective study highlighting whether patients’ characteristics and surgeons’ prior experience could impact the conversion.

## METHODS

From January 2010 to December 2020, appendectomy for acute appendicitis was performed on 853 patients in the Surgical Department at Habib Thameur Hospital in Tunis, Tunisia. Of these, 725 (84.9%) underwent LA. The clinical, demographic, surgical, and pathological data of these patients were included in a prospective database and were retrieved from our hospital. To record, only LA were considered in the analysis.

The following factors were analyzed to determine which ones were related to the conversion from LA to OA: age, sex, body mass index (BMI), previous abdominal surgery, comorbidities, clinical and laboratory parameters including Alvarado score^1^, preoperative C-reactive protein (CRP), intraoperative ﬁndings such as anatomy and degree of inﬂammation.

During the study period, LA were performed by different surgeons both resident and attending surgeons. The decision to convert to an open procedure was made personally by the surgeon.

### Inclusion criteria

The inclusion criteria were: all adult patients older than 14 years of age, admitted with acute appendicitis and who underwent LA.

### Exclusion criteria

The exclusion criteria were: patients with missing data, incomplete records, those who underwent two simultaneous procedures, and direct OA cases.

### Statistical analysis

All data were assessed with the Statistical Package for Social Sciences (SPSS) software (version 25.0). Multivariate analysis was performed with logistic regression. Two-sided p-values <0.05 were considered statistically signiﬁcant. The qualitative variables were expressed by their percentages, the quantitative variables by the mean, and by standard deviation (±SD) when the distribution was Gaussian, otherwise, by the median, interquartile, and extremes. Once the data analyzed were from the hospital database, prior approval of the Institution’s Ethics Committee was waived.

### The technique of laparoscopic appendectomy

LA was performed using a 3-trocar approach (umbilical, 10 mm port; suprapubic, 5 mm port; and lower-left quadrant, 10 mm port), in the Trendelenburg position.

The appendix was extracted through the lower-left quadrant trocar in a plastic bag or through the trocar when removed, depending on the operator preference.

The technique of OA was performed via Mc-Burney’s incision or midline incision.

## RESULTS

The study enrolled 725 patients who underwent LA. Demographic, clinical, and pathological data of the patients included in the analysis are summarized in Tables 1 and 2.

**Table 1:** Patients’ demographics.

		n (%)
Sex		
	Male	201 (27.7)
	Female	524 (72.3)
Age (mean±SD)		35.2±16
Comorbidities		
	One or more	40 (5.5)
	None	685 (94.5)
Type of comorbidities		
	Arterial hypertension	7 (0.97)
	Cardiovascular morbidities	6 (0.83)
	Chronic obstructive pulmonary disease	3 (0.42)
	Diabetes	24 (3.31)
BMI (kg/m^2^) mean±SD		24.6±17.8
Previous abdominal surgery		36 (4.97)
Tenderness of iliac fossa		596 (82.2)
Defense of iliac fossa		76 (10.5)
Diffuse tenderness		20 (2.76)
Nausea and/or vomiting		324 (44.7)
Fever		336 (46.34)
WBC count (>10.000/mm^3^)		572 (78.9)
Alvarado score (mean±SD)		5.4±1.7
CRP (mg/dL) (mean±SD)		44±162.59

BMI: body mass index; WBC: white blood cell; SD: standard deviation; CRP: C-reactive protein.

**Table 2:** Surgical and anatomical characteristics.

		n (%)
Surgeon		
	Attending surgeon	225 (31)
	Resident	500 (69)
Retrocecal appendix		112 (15.45)
Edematous inflammation		193 (26.62)
Phlegmonous inflammation		168 (23.17)
Gangrenous inflammation		132 (18.2)
Perforated appendicitis		59 (8.14)
Appendicular abscess		320 (44.14)
Diffuse peritonitis		24 (3.31)
Conversion		121 (16.7)
Cause of conversion:		
	Difficult dissection	351 (48.4)
Surgery time (minutes, mean±SD)		69.5±25.1

SD: standard deviation.

**Figure 1:**
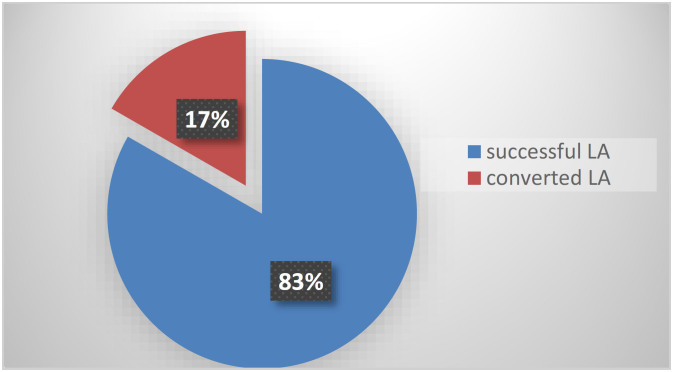
Overall laparoscopic appendectomy conversion rate and successful completed laparoscopic rate. LA: laparoscopic appendectomy.

A total of 604 (83.3%) procedures were successfully performed using the laparoscopic approach while 121 (16.7%) were converted to the open approach (Figure 1). The mean age was 35.2±16 years with a range from 15–80 years. LA patients were signiﬁcantly younger than the OA (34±14 vs 48±16), which is statistically signiﬁcant (p<0.01).

LA was completed without conversion in 147 male patients and 457 female patients. The ratio of females to males is almost 2.6.

Previous abdominal surgery in LA group was performed in 32/604 (5.3%) patients, and in OA, it was performed in 4/121 (3.3%).

On comparing the clinical presentation of appendicitis, fever was the most common symptom, followed by vomiting and diarrhea in both groups. Symptoms of diffuse tenderness, rebound tenderness, localized guarding, and diffuse guarding were found to be signiﬁcantly associated with conversion appendicectomy.

The preoperative CRP was higher in the OA group (162.59±100.4) with p<0.001, which is statistically signiﬁcant. The present study highlights that significantly elevated CRP is also an independent risk factor for conversion from LA to OA.

The computed tomography (CT) scan ﬁndings showed that the presence of signiﬁcant fat stranding, free ﬂuid or free air, abscess formation, and CT grade of 4–5 signiﬁcantly increased the possibility of conversion; in our study, we found that 68 patients were converted due to the presence of an abscess.

Intraoperatively, diﬃcult dissection associated with a severe acute inﬂammatory process has been the most common reason to convert (84/121; 69.4%), followed by perforated appendix (44/121; 36.36%), and retrocecal appendix (44/121; 36.36%).

Other reasons justiﬁed the conversion, such as diﬃculty to identify the appendix, uncontrolled bleeding, impossibility to maintain an adequate pneumoperitoneum, and hypotension due to the Trendelenburg’s position.

The univariate analysis of factors related to conversion is shown in Tables 3 and 4, concluding that diffuse tenderness is a signiﬁcant predictive factor for the conversion.

**Table 3:** Univariate analysis on demographics and clinical factors related to conversion.

		LA group n=604 n (%)	OA group n=121 n (%)	p-value
Sex				0.003
	Male	145 (24)	54 (44.6)	
	Female	457 (76)	67 (55.4)	
Age (mean±SD)		34±14	48±16	0.001
Comorbidities				0.760
	One or more	32 (5.3)	8 (6.6)	
	None	572 (94.7)	113 (93.4)	
Type of comorbidities				
	Arterial hypertension	3 (0.5)	4 (3.3)	0.017
	Cardiovascular morbidities	2 (0.33)	4 (3.3)	0.008
	Chronic obstructive pulmonary disease	1 (0.16)	2 (1.65)	0.074
	Diabetes	20 (3.31)	4 (3.3)	0.740
BMI (kg/m^2^, mean±SD)		23.5±4.3	25.8 ± 4.9	0.716
Previous abdominal surgery		32 (5.3)	4 (3.3)	0.469
Tenderness of iliac fossa		524 (86.75)	72 (59.5)	0.455
Defense of iliac fossa		44 (7.28)	32 (26.45)	0.612
Diffuse tenderness		8 (1.32)	12 (9.92)	0.009
Nausea and/or vomiting		184 (30.46)	140 (1.16)	0.703
Fever		276 (45.7)	60 (49.6)	0.670
WBC count (>10.000/mm^3^)		530 (87.7)	42 (34.7)	0.568
Alvarado score (mean±SD)		5.3±1.6	5.5±1.9	0.0025
CRP (mg/dL) (mean±SD)		44±51.04	100.4±162.59	<0.001

CRP: C reactive protein; SD: standard deviation; WBC: white blood cell; BMI: body mass index; LA: laparoscopic appendectomy; OA: open appendectomy.

**Table 4:** Univariate analysis on surgical and pathological factors related to conversion.

		LA n (%)	OA n (%)	p-value
Surgeon				1
	Attending surgeon	117 (19.4)	108 (89.26)	
	Resident	487 (80.6)	13 (10.74)	
Retrocecal appendix		68 (11.26)	44 (36.36)	<0.001
Phlegmonous inflammation		148 (24.5)	20 (16.53)	1
Perforated appendicitis		15 (12.4)	44 (36.36)	<0.001
Appendicular abscess		289 (47.8)	31 (25.6)	0.001
CRP (mg/dL)		44±51.04	100.4±162.59	<0.001
Difficult dissection		267 (44.2)	84 (69.4)	<0.001

LA: Laparoscopic appendectomy; OA: open appendectomy; CRP: C-reactive protein.

A multivariate analysis (Table 5) was then conducted to assess which of the factors were independently related to the decision to convert the intervention. The factors statistically predictive of conversion were: presence of comorbidities (odds ratio [OR] 3.1; 95% confidence interval [CI] 1.1–8.8; p<0.029), a ﬁnding of an appendiceal perforation (OR 5.2; 95%CI 1.8–15.0; p<0.003), retrocecal appendix (OR 5.0; 95%CI 1.7–14.8; p<0.004), gangrenous appendix, the presence of appendicular abscess (OR 3.6; 95%CI 1.2–11.1; p<0.023) and the presence of diﬃcult dissection (OR 9.2; 95%CI 1.8–47.8; p<0.008).

**Table 5:** Multivariate analysis of factors related to conversion.

Factors	OR (95%CI)	p-value
Comorbidities	1.27 (0.57–2.83)	0.029
Male/Female	2.54 (1.7–3.81)	0.295
Appendiceal perforation	5.2 (1.8–15)	0.003
Retrocecal appendix	4.5 (2.87–13.8)	0.004
Appendicular abscess	3.3 (1.2–11)	0.023
Alvarado score^1^ (mean±SD)	1.329 (1.024–1.724)	0.033
Difficult dissection	2.87 (2.56–3.2)	0.045
CRP	2.69 (0.989–1.16)	0.002

SD: standard deviation; OR: odds ratio; CI: confidence interval; CRP: C-reactive protein.

## DISCUSSION

Since its initial description by Kurt Karl Stephan Semm, many other studies had encouraged LA over OA, due to its advantages^12,15^. LA leads to lower postoperative pain, shorter postoperative hospital stay, conducting to a reduced recovery time^7,11,14,15^.

Conversion rate is one of the main factors used to measure laparoscopic competence^5^. The rate of conversion evaluated by many studies was related to various factors and was more associated with higher hospital costs^10^. The main goal of this study was to develop a clinical tool that enables surgeons to estimate the risk of conversion from AL to OA based on objective and easily available preoperative patient parameters.

Almost all studies on the conversion rate from LA to OA affirmed a rate of 9–12%^8,13^.

The conversion rate of 16.7% in this current work is almost identical to the literature’s results. We concluded that multiple factors related to patients’ characteristics may inﬂuence LA conversion to OA^12^ such as advanced age (≥65)^10,12^; patients older than 65 years were associated with higher conversion rate (33.3%) compared to <65 years-old patients (8.9%), (p-value 0.002)^10^. In our study, advanced age was associated with a higher rate of conversion (p-value 0.001) as well.

Other studies demonstrated that males and old age were signiﬁcant independent factors predicting conversion to OA^4^. Male patients consulting for right lower abdominal pain were more associated with perforated appendicitis, thereby they had the highest-level conversion^2,11,12^.

The majority of patients (96.7%) who underwent laparoscopic conversion recorded in this study, had no previous abdominal surgery. However, the most common reason of conversion was a severe inﬂammation causing diﬃcult and unsafe dissection. Delayed presentation of appendicitis is also associated with a higher risk of conversion to OA^4^.

On physical examination, diffuse tenderness carried a high risk of conversion to OA (9.92%; p<0.009)^11^, whereas in CT scan, severe inﬂammation was considered to be an independent predictor of conversion^4^.

Peedikathara et al.^11^ found that ultrasonography ﬁndings like an abscess (OR -8.000; 95%CI -1.006–63.962; p=0.049) and probe tenderness without visualizing appendix (OR -0.133; 95%CI -0.020–0.880; p=0.036) were signiﬁcantly predicting factors of conversion to OA.

The CT scan grade of 4–5 is an important predictor factor of conversion^10^. In our study, the Alvarado score^1^ is signiﬁcantly associated with a higher rate of conversion (p<0.0025).

The CRP is a good marker of severe inﬂammation which could be a signiﬁcant risk factor for conversion to OA. A higher value of CRP is associated with severe located inﬂammatory reaction with increased risk of bleeding, adhesion with the surrounding tissue, and complicated appendicitis^4^.

According to the receiver operating characteristic (ROC) analysis of Aydın et al.^2^, a CRP value of 108.5 mg/L was a risk factor for the conversion to OA. Shimoda et al.^13^ concluded that preoperative CRP of >100 and >150 mg/L were a statistically signiﬁcant predictor for conversion.

Increased value of white blood cell (WBC) count >15000 cells/cumm was seen to be more associated with conversion to OA, but it is not a statistical independent factor related to conversion^2^. Aydın et al. indicated that a neutrophil ratio of 81.5% was a signiﬁcant predictive factor of a higher rate conversion because it concludes to bacterial translocation leading to diffuse peritonitis and intraabdominal abscess development^2^.

The preoperative ﬁndings such as severe inﬂammation, gangrenous and perforated appendicitis are the major factors impacting the operative diﬃculties and associated with conversion to OA^6,8,13^.

Wei et al.^14^ concluded to higher conversion rate due to less surgeon experience. Inexperienced surgeons couldn’t handle the intraoperative diﬃculties leading usually to conversion to OA^12^. Liu et al.^10^ have conﬁrmed this relationship between the higher rate of conversion (31.3%) and the inexperienced surgeons (≤10 LA performed). However, in the present study, there was no difference in the overall conversion rate between experienced surgeons and resident surgeons, which can be explained by their good initial learning curve.

The higher surgical level of experience in LA leads to a shorter operating time, especially in complicated appendicitis which can need hand touching^6^. Intra-operative complications like Iatrogenic bowel injury and bleeding could not be managed laparoscopically, and mostly lead to open conversion^16^.

This study had concluded to independent factors of conversion: advanced age, Alvarado score^11^, high CRP value, and intraoperatively ﬁndings such as retrocecal appendix, perforated appendicitis, and appendicular abscess.

## CONCLUSIONS

Laparoscopic appendectomy is a minimally invasive procedure that has become recommended as a standard technique for routine appendicitis, resulting in a better cosmetic outcome, and a shorter recovery time. Conversion from LA to OA procedure is done for more than one reason. Preoperatively, it is possible to identify predictive factors for conversion to laparotomy, and the ability to identify these reasons of conversion can aid surgeons in selecting patients who would beneﬁt from a primary open appendectomy.

## References

[1] Antonacci N, Ricci C, Taffurelli G, Monari F, Del Governatore M, Caira A (2015). Laparoscopic appendectomy: which factors are predictors of conversion? A high-volume prospective cohort study. Int J Surg.

[2] Aydın HO, Avcı T, Tezcaner T, Kırnap M, Yıldırım S, Moray G (2018). Role of preoperative C-reactive protein value and neutrophil ratio in the determination of conversion from laparoscopic appendectomy to open appendectomy. Ulus Travma Acil Cerrahi Derg.

[3] Belotto M, Coutinho L, Pacheco AM, Mitre AI, Fonseca EAD (2022). Influence of minimally invasive laparoscopic experience skills on robotic surgery dexterity. Arq Bras Cir Dig.

[4] Choi KW, Park BK, Suh SW, Lee ES, Lee SE, Park JM (2019). Risk factors for additional port insertion in single-port laparoscopic appendectomy. Wideochir inne tech maloinwazyjne.

[5] Deboni VS, Rosa MI, Lima AC, Graciano AJ, Garcia CE (2022). The appendicitis inflammatory response score for acute appendicitis: is it important for early diagnosis?. Arq Bras Cir Dig.

[6] Finnerty BM, Wu X, Giambrone GP, Gaber-Baylis LK, Zabih R, Bhat A (2017). Conversion-to-open in laparoscopic appendectomy: a cohort analysis of risk factors and outcomes. Int J Surg.

[7] Hansen JB, Smithers BM, Schache D, Wall DR, Miller BJ, Menzies BL (1996). Laparoscopic versus open appendectomy: prospective randomized trial. World J Surg.

[8] Hiramatsu K, Toda S, Tate T, Fukui Y, Tomizawa K, Hanaoka Y (2018). Can laparoscopic appendectomy be safely performed by surgical residents without prior experience of open appendectomy?. Asian J Surg.

[9] Korndorffer Jr, Fellinger E, Reed W (2010). SAGES guideline for laparoscopic appendectomy. Surg Endosc.

[10] Liu SI, Siewert B, Raptopoulos V, Hodin RA (2002). Factors associated with conversion to laparotomy in patients undergoing laparoscopic appendectomy. J Am Coll Surg.

[11] Peedikathara LM, Mandumpala JM, Vallon SM, Kavalakat AJ (2018). Predictors for conversion to open appendicectomy in patients undergoing laparoscopic appendicectomy: a prospective study. Int Surg J.

[12] Sakpal SV, Bindra SS, Chamberlain RS (2012). Laparoscopic appendectomy conversion rates two decades later: an analysis of surgeon and patient-specific factors resulting in open conversion. J Surg Res.

[13] Shimoda M, Maruyama T, Nishida K, Suzuki K, Tago T, Shimazaki J (2019). Preoperative high C-reactive protein level is associated with an increased likelihood for conversion from laparoscopic to open appendectomy in patients with acute appendicitis. Clin Exp Gastroenterol.

[14] Wei HB, Huang JL, Zheng ZH, Wei B, Zheng F, Qiu WS (2010). Laparoscopic versus open appendectomy: a prospective randomized comparison. Surg Endosc.

[15] Yau KK, Siu WT, Tang CN, Yang GP, Li MK (2007). Laparoscopic versus open appendectomy for complicated appendicitis. J Am Coll Surg.

[16] Yu MC, Feng YJ, Wang W, Fan W, Cheng HT, Xu J (2017). Is laparoscopic appendectomy feasible for complicated appendicitis? A systematic review and meta-analysis. Int J Surg.

